# Modulation of Gut Microbiota for Health by Current and Next-Generation Probiotics

**DOI:** 10.3390/nu11081921

**Published:** 2019-08-15

**Authors:** Reetta Satokari

**Affiliations:** Human Microbiome Research Program, Faculty of Medicine, University of Helsinki, FI-00014 Helsinki, Finland; reetta.satokari@helsinki.fi; Tel.: +358-40-821-2919

**Keywords:** probiotic, commensal bacteria, gut microbiota, human health

## Abstract

The human gut microbiota is a complex ecosystem and has an essential role in maintaining intestinal and systemic health. Microbiota dysbiosis is associated with a number of intestinal and systemic conditions and its modulation for human health is of great interest. Gut microbiota is a source of novel health-promoting bacteria, often termed as next-generation probiotics in order to distinguish them from traditional probiotics. The previous lessons learned with traditional probiotics can help the development of next-generation probiotics that target specific health issues and needs.

Human intestinal microbiota is a highly diverse population of microbes consisting of bacteria, archaea, fungi, viruses, and protozoa, and it is considered to have a major contribution to human health [1]. Microbiota alterations in the composition, diversity, and temporal stability (microbiota dysbiosis) have been associated with a number of gastrointestinal (GI) and systemic conditions [1]. Therefore, modulation of the intestinal microbiota to maintain a favorable balance in the ecosystem and to improve human health is of great interest.

The review “Effects of Probiotics, Prebiotics, and Synbiotics on Human Health” [2] summarizes the evidence and discusses the possibilities of influencing gut and systemic health with nutritional approaches using probiotics, prebiotics, and their combinations (synbiotics). Probiotics are defined as “live strains of strictly selected microorganisms that, when administered in adequate amounts, confer a health benefit on the host”. The most commonly used probiotic strains belong to the genera *Lactobacillus and Bifidobacterium*, but also *Lactococcus*, *Streptococcus*, *Enterococcus*, and *Bacillus* spp. and some yeast strains belonging to the genus *Saccharomyces* have been included in probiotic products for human nutrition [2]. The use of probiotics in food products is based on their safety records for human use, and most of the used species have Generally Regarded as Safe (GRAS; FDA, US) or Qualified Presumption of Safety (QPS; EFSA, EU) status [2]. Prebiotics are defined as nonviable food components that confer a health benefit on the host which is associated with modulation of the microbiota. Nondigestible oligosaccharides such as fructo- and galactooligosaccharides and inulin (a fructopolysaccharide) are among the most studied prebiotics [2].

Based on the extensive summary of probiotic clinical trials by Markowiak and Śliżewska [2], it seems that probiotics have clinical benefits especially in populations that are at risk of developing a disease, but more clinical investigations should be done to verify the efficacy. The authors underline that the effects may depend on the strain, dose, and components used to produce a given probiotic product [2]. Endogenous microbiota adds yet another factor that can have an impact on the probiotic efficacy at the individual level, as it may impact colonization and functionality of the administered strain. In general, orally consumed probiotic *Lactobacillus* and *Bifidobacterium* strains colonize the gut only transiently and disappear from feces within days or weeks after the administration [3]. However, a recent study showed that long-term persistence of an administered *Bifidobacterium longum* strain for up to six months is possible in a subset of individuals and that the colonization was more efficient when endogenous *B. longum* strains were underrepresented [4]. Thus, the engraftment was influenced by the endogenous microbiota and a niche opportunity in the gut ecosystem. In this respect, the colonization of probiotics in infants with undeveloped microbiota and weaker colonization resistance may be more long-term, possibly even for a lifetime, and the health effects may also be more profound, which emphasizes the importance of appropriate strain selection. 

The traditional selection criteria for probiotics take into account both safety and functionality as well as technological usefulness [2]. In addition to the selection criteria summarized in [2], probiotic products may need to be tailored for the specific target subpopulation of interest. A recently reported study used such an approach, where the authors first carried out a pilot study and selected the probiotic strain to be used by its ability to effectively colonize in the target population, and then demonstrated in a large clinical trial (*n* = 4556) that a synbiotic mix containing the selected strain *Lactobacillus plantarum* and prebiotic fructooligosaccharides reduced neonatal sepsis and death among infants in rural India by 40 % [5].

While probiotics offer an attractive preventive or adjunct therapy for various diseases [2], the most striking evidence of the possibilities to treat diseases by microbiota modulation has been obtained by fecal microbiota transplantation (FMT) treatment of recurrent *Clostridioides difficile* (rCDI) infection, with efficacy rates even exceeding 90% [6]. In rCDI patients, microbiota modulation by FMT has been shown to restore the disturbed ecosystem and the effect is long-term [7,8]. Thereby, FMT is currently considered as a possible treatment option for several diseases that are associated with microbiota dysbiosis [6]. Gut microbiota research and FMT studies have underlined the importance of commensal species in maintaining gut health and provided leads for the development of next-generation probiotics and live biotherapeutics based on core species, i.e., commensals that are underrepresented in microbiota dysbiosis [9,10]. On the other hand, prebiotics have great potential in modifying the residing gut microbiota and stimulating beneficial bacteria therein, and their potential should be studied further [2]. For example, the consumption of dietary fiber or prebiotics such as inulin may increase the abundance of *Faecalibacterium prausnitzii* [11], which is an abundant anti-inflammatory gut species and is found to be depleted in individuals with inflammatory bowel disease [12]. In general, prebiotic-based nutritional approaches seem very attractive in targeting extremely oxygen-sensitive commensals such as *F. prausnitzii*, whose utilization as a next-generation probiotic could prove technologically very challenging.

Adherence to the intestinal epithelium and immunomodulation have been considered as desired probiotic properties [2], and screening for such properties may prove useful also for the strain selection of next-generation probiotics. Further selection properties may include also the production of extracellular vesicles (EV), which due to their small size can reach the gut epithelium in places where the mucus layer is firm and impermeable to bacteria and enable bacteria–host crosstalk without direct contact. Both Gram-negative and Gram-positive bacteria, including several *Lactobacillus* and *Bifidobacterium* strains, have been found to produce EVs, which can carry different components and functional cargos depending on the organism and growth conditions [13]. Importantly, many promising next-generation probiotics candidates, such as *Bacteroides* spp. and *Akkermansia muciniphila*, are known to produce EVs, which may contribute to their functionality in alleviating inflammation and reinforcing epithelial integrity [10,13,14]. EVs could possibly be used as biotherapeutic agents to avoid the risks associated with the use of live bacteria in high-risk individuals, e.g., those with compromised immunity.

The knowledge on microbes with potential health benefits has extended dramatically in the past decade. Gut microbiota is a source of novel probiotics and prebiotic innovations, and with the introduction of next-generation probiotics, we are entering a new era of probiotic research. In the future, we are likely to see probiotics that have been developed to target specific health issues and needs. In this regard, a proof-of-concept study with *A. muciniphila* supplementation showed an improvement of metabolic parameters in overweight and obese individuals [15]. The translation of gut microbiota research results to human health has begun, but many of these are still at the very early stage of investigation, and the lessons learned from traditional probiotics can help the development of next-generation probiotics.

## References

[1] Lavelle A., Hill C. (2019). Gut Microbiome in Health and Disease: Emerging Diagnostic Opportunities. Gastroenterol. Clin. North Am..

[2] Markowiak P., Śliżewska K. (2017). Effects of Probiotics, Prebiotics, and Synbiotics on Human Health. Nutrients.

[3] Mättö J., Fondén R., Tolvanen T., Von Wright A., Vilpponen-Salmela T., Satokari R., Saarela M. (2006). Intestinal survival and persistence of probiotic Lactobacillus and Bifidobacterium strains administered in triple-strain yoghurt. Int. Dairy J..

[4] Maldonado-Gómez M.X., Martínez I., Bottacini F., O’Callaghan A., Ventura M., van Sinderen D., Hillmann B., Vangay P., Knights D., Hutkins R.W. (2016). Stable Engraftment of Bifidobacterium longum AH1206 in the Human Gut Depends on Individualized Features of the Resident Microbiome. Cell Host Microbe.

[5] Panigrahi P., Parida S., Nanda N.C., Satpathy R., Pradhan L., Chandel D.S., Baccaglini L., Mohapatra A., Mohapatra S.S., Misra P.R. (2017). A randomized synbiotic trial to prevent sepsis among infants in rural India. Nature.

[6] Allegretti J.R., Mullish B.H., Kelly C., Fischer M. (2019). The evolution of the use of faecal microbiota transplantation and emerging therapeutic indications. Lancet.

[7] Jalanka J., Mattila E., Jouhten H., Hartman J., de Vos W.M., Arkkila P., Satokari R. (2016). Long-term effects on luminal and mucosal microbiota and commonly acquired taxa in faecal microbiota transplantation for recurrent Clostridium difficile infection. BMC Med..

[8] Fuentes S., van Nood E., Tims S., Heikamp-de Jong I., ter Braak C.J., Keller J.J., Zoetendal E.G., de Vos W.M. (2014). Reset of a critically disturbed microbial ecosystem: Faecal transplant in recurrent Clostridium difficile infection. ISME J..

[9] O’Toole P.W., Marchesi J.R., Hill C. (2017). Next-generation probiotics: The spectrum from probiotics to live biotherapeutics. Nat. Microbiol..

[10] Hiippala K., Jouhten H., Ronkainen A., Hartikainen A., Kainulainen V., Jalanka J., Satokari R. (2018). The Potential of Gut Commensals in Reinforcing Intestinal Barrier Function and Alleviating Inflammation. Nutrients.

[11] Verhoog S., Taneri P.E., Díaz Z.M.R., Marques-Vidal P., Troup J.P., Bally L., Franco O.H., Glisic M., Muka T., Díaz Z.R. (2019). Dietary Factors and Modulation of Bacteria Strains of Akkermansia muciniphila and Faecalibacterium prausnitzii: A Systematic Review. Nutrients.

[12] Sokol H., Pigneur B., Watterlot L., Lakhdari O., Bermudez-Humaran L.G., Gratadoux J.J., Blugeon S., Bridonneau C., Furet J.P., Corthier G. (2008). Faecalibacterium prausnitzii is an anti-inflammatory commensal bacterium identified by gut microbiota analysis of Crohn disease patients. Proc. Natl. Acad. Sci. USA.

[13] Molina-Tijeras J.A., Gálvez J., Rodríguez-Cabezas M.E. (2019). The Immunomodulatory Properties of Extracellular Vesicles Derived from Probiotics: A Novel Approach for the Management of Gastrointestinal Diseases. Nutrients.

[14] Cani P.D., de Vos W.M. (2017). Next-Generation Beneficial Microbes: The Case of Akkermansia muciniphila. Front. Microbiol..

[15] Depommier C., Everard A., Druart C., Plovier H., Van Hul M., Vieira-Silva S., Falony G., Raes J., Maiter D., Delzenne N.M. (2019). Supplementation with Akkermansia muciniphila in overweight and obese human volunteers: A proof-of-concept exploratory study. Nat. Med..

